# Supplementary material for: Pharmacokinetics, feasibility and safety of co-administering azithromycin, albendazole, and ivermectin during mass drug administration: A review
Source: PLoS Negl Trop Dis. 2023 Jun 14;17(6):e0011224. doi: 10.1371/journal.pntd.0011224 (PMC10298764; doi:10.1371/journal.pntd.0011224)
Supplement: Attachment — Submitted filename: Literature review_comments.docx [file pntd.0011224.s001.docx]

24^th^ May 2023

Dear PLOS NTDs,

Re: PNTD-D-23-00311
*Pharmacodynamics, feasibility and safety of co-administering azithromycin, albendazole, and ivermectin during mass drug administration: a review*

Many thanks for provisionally accepting our paper for publication in PLOS Neglected Tropical Diseases and for the extremely helpful comments of the peer reviewers. We have provided a point-by-point response below and attach an updated manuscript with track changes and a clean copy.

We look forward to hearing from you,

Best Wishes,

Michael Marks & Scott McPherson

On Behalf of the Authors

**Reviewer #1:**

Since there are no pharmacodynamic analyses reviewed or presented, the title should be revised accordingly.

Thanks very much for this point. We’ve revised to Pharmacokinetics given the papers found relating to that topic.

Similarly, in the methods section of the abstract and body of the manuscript, the word “dynamics” should be deleted. Thank you.

This has been deleted.

The pharmacokinetic studies cited were conducted in adults. The authors should indicate whether studies in the pediatric population would be necessary, or whether the results in adults could be extrapolated to the pediatric population.

Thanks very much. The following has been added to the discussion section. “None of the relevant papers included a pediatric population. While the available research suggests extrapolation of results from adults to children may be possible, further studies specifically focused on children would be an advantage for large scale coadministration expansion.”

Please double check reference 11.

Chuck Knirsch has been inadvertently been left off the reference -we have corrected this.

Although the conclusion is based on triple-drug combination, the authors failed to capture a key component of confidence in safety; that is, the number of exposures over time for each individual component of the combination. Ivermectin has been used widely in MDA since 1989 alone or in combination with albendazole. Albendazole has been widely used since the 1980s in community-based treatment paradigms either alone or in combination with other drugs. Azithromycin is a widely used antibiotic for multiple indications that has also been used in trachoma MDA campaigns. Although there may be a paucity of information on the three drugs used in combination as single doses, our confidence in safety is large given the millions of doses administered worldwide over the last 30+ years. The same would not be true for new chemical entities used in combination. I would suggest that the authors revisit their discussion about confidence in safety along these lines.

Thank you very much. The following has been added to the intro section to capture this reflection with reference:

Confidence in the safety of these drugs combined is bolstered by a long history of mass drug administration among endemic communities. Ivermectin has been consistently used at scale since the Mectizan Donation Program began donating Ivermectin in 1987 (1) administered alone or together with albendazole. Azithromycin is a commonly used antibiotic that has also been used in trachoma MDA campaigns throughout the world. Collectively many hundred million doses of each agent have been delivered as part of NTD programmes.

With regard to pharmacokinetics, the authors cite the relevant studies available in the scientific literature. Having said that, there was mention of the analyte albendazole but not the active metabolite albendazole sulfoxide. Albendazole administered orally is poorly bioavailable, partly through pH-dependent solubility mitigated in part by the administration of food, but also because albendazole parent is rapidly metabolized (likely in both gut and liver by CYP3A4) to albendazole sulfoxide. In fact, following a single 400 mg dose of albendazole, it is difficult to measure albendazole parent and the bioactive metabolite is indeed the more important analyte. In one of the pharmacokinetic studies cited (Amsden et al), there was virtually no change in albendazole parent when administered with azithromycin, whereas there was a decrease in albendazole sulfoxide when administered in combination. Although the decrease is not likely clinically relevant, it illustrates the point thatone should take into account the relevant analyte, in this case the active metabolite of this anthelminthic drug albendazole.

The following has been added into the pharmacokinetic section with reference: Albendazole sulfoxide, the active metabolite after absorption, decreased when both drugs were administered together compared to when albendazole was administered alone.

There is a known food effect for both albendazole and ivermectin exposures. The authors should take this into account when reviewing the pharmacokinetic and safety data and discuss whether food should be recommended when the three drugs are used in combination.

Thanks very much. The following has been added to the Discussion section:

Given the relative a paucity of data on the co-administration of these three drugs, it may also be relevant to also investigate whether administration with or without food further impacts drug levels.

I would agree with the authors that there is unlikely to be a clinically-relevant drug-drug pharmacokinetic interaction with albendazole, ivermectin and azithromycin in combination. Based on the wide-use of these drugs individually, in two-drug combinations (ie ivermectin and albendazole), or in triple drug combination where yaws or trachoma are prevalent, the likelihood of a significant novel safety finding – especially with regard to serious adverse events – is unlikely to surface. Having said that, if the triple combination is being considered for WHO guidelines review, the standard practice has been to study the combination in ~10,000 subjects to rule out rare serious adverse events that could be missed in studies with small sample sizes. Such was done recently in lymphatic filariasis when IDA (ivermectin-diethylcarbamazine-albendazole) was being considered and the process has been described: Weil GJ, Fischer PU, Krentel A. Lessons from Large-Scale Tolerability and Acceptability Studies of Triple Drug Mass Drug Administration Performed to Support Policy Change and Accelerate Elimination of Lymphatic Filariasis. Am J Trop Med Hyg. 2022 Mar 15;106(5_Suppl):13-17. Perhaps the authors could comment on the target policy profile of ivermectin-albendazole-azithromycin and whether a more formal assessment should be undertaken for this combination. Thanks very much.

Thanks very much. We’ve included the following in the Discussion section with a reference to the study you’ve referenced: Given the ultimate goal of trials involving the co-administration of these drugs, the studies found in the existing literature have not achieved the sample size that WHO has previously used for guideline adoption in similar co-administration trials of different drug combinations.(24)

**Reviewer #2:**

Major comments:
1.       Pharmacodynamics in the title.  The manuscript focuses more on safety, side effects and pharmacokinetic drug interactions.  Pharmacodynamics is not a major focus of the manuscript and I would recommend removal from the title. Thanks very much. This has been changed to Pharmacokinetics.

2.      It is not clear what side affects were evaluated.  It would be useful to describe the side effects reported/evaluated if there were consistent across studies. Perhaps listing major side effects and frequency in tabular form would be helpful.

As noted in the limitations section one of the issues has been the non-standard way in which adverse events have been recorded in previous studies. As this was already included in the limitations we have not made any further changes on this point.

3.      There is no dosing information provided.  It would be useful to include doses throughout, or perhaps a table with dose information included.

We have ensured that the doses are given for each study throughout.
